# Preoperative plateletcrit is a Prognostic Biomarker for Survival in Patients with Non-Small Cell Lung Cancer

**DOI:** 10.7150/jca.41122

**Published:** 2020-02-25

**Authors:** Joon Young Hur, Ha Yeon Lee, Hye Jung Chang, Cheon Woong Choi, Dae Hyun Kim, Wan Kyu Eo

**Affiliations:** 1 Division of Hematology and Oncology, Department of Medicine, Samsung Medical Center, Sungkyunkwan University School of Medicine, Seoul, Korea.; 2 Division of Hematology and Oncology, Department of Internal Medicine, National Medical Center, Seoul, Korea.; 3 Department of Internal Medicine, Kyung Hee University Hospital at Gangdong, Seoul, Korea; 4 Department of Thoracic Surgery, Kyung Hee University Hospital at Gangdong, Seoul, Korea

**Keywords:** plateletcrit, non small cell lung cancer, mean platelet volume.

## Abstract

**Objectives:** Several factors associated with the prognosis of patients with NSCLC have been reported in the literature; however, most of these factors cannot be examined preoperatively. In this study, the clinical utility of platelet parameters in patients with NSCLC who underwent curative resection was evaluated.

**Materials and Methods:** A retrospective study on patients with NSCLC who underwent curative resection from July 2006 to September 2016 was conducted. The Cox proportional hazard regression model was applied to evaluate the variables that demonstrated effects on disease-free and overall survival (DFS and OS).

**Results:** A total of 116 patients with NSCLC were analyzed. There were 15 patients with plateletcrit greater than 0.2755%, and 101 patients whose plateletcrit was 0.2755% or lower. Multivariate analysis identified plateletcrit higher than 0.2755% (hazard ratio [HR] = 4.18, 95% confidence interval [CI] = 1.54-11.34, P =0.004), patient age of 65 years or more (HR = 4.02, 95% CI = 1.67-9.66, P = 0.001), and stage II or IIIA disease (HR = 2.95, 95% CI = 1.26-6.87, P = 0.012) as independent factors for OS that predicted a poor prognosis. Multivariate analysis identified plateletcrit higher than 0.2755% (HR = 4.07, 95% CI = 1.52-10.94, P = 0.005), stage II or IIIA disease (HR = 5.38, 95% CI = 2.71-10.66, P < 0.001) and non-adenocarcinoma (HR = 1.92, 95% CI = 1.02-3.59, P = 0.040) as independent prognostic factors for DFS that predicted a poor prognosis.

**Conclusion:** Our results suggest a potential role of preoperative plateletcrit as an independent prognostic marker for patients with resectable NSCLC.

## Introduction

Lung cancer is the leading cause of cancer-related death in men and women in the United States 1 and South Korea 2. Lung cancers are classified into small cell or non-small cell types, for the purpose of treatment. Non-small cell lung cancers (NSCLCs) account for approximately 80% of all lung cancers 3. Most lung cancers (57%) are diagnosed at advanced stages, because early disease is typically asymptomatic; only 16% of cases are diagnosed early in the disease process 4. For the treatment of stage I and II NSCLC, most patients (69%) undergo surgery, and about 25% of these also receive additional chemotherapy or radiation therapy, or both 4. On the other hand, most patients with stage III and IV NSCLC (53%) receive chemotherapy, with or without radiation therapy 4.

In patients with advanced or relapsed lung cancers, testing for the presence of epidermal growth factor receptor (EGFR)-sensitizing mutations 5 and the status of anaplastic lymphoma kinase (ALK) gene rearrangements prior to the use of tyrosine kinase inhibitors (TKIs) for therapy has substantially improved survival outcomes 6. Recently, programmed death ligand 1 (PD-L1) testing has been included in the evaluation of primary NSCLC, in accordance with the National Comprehensive Cancer Network guidelines (2017 version). Immunotherapeutic drugs that act by targeting the programmed cell death receptors on T cells prolong the disease-free periods and overall survival (OS) in patients with advanced NSCLC 7; further studies on these aspects in patients with early disease are ongoing 8. Overexpression of p53 protein is prognostic for poorer survival and is predictive of differentially greater survival benefits from receiving adjuvant chemotherapy 9. Several factors associated with the prognosis of patients with NSCLC have been reported in the literature; however, most of these factors cannot be examined preoperatively 3 .

In terms of laboratory factors, an increased systemic inflammatory response (SIR) has been demonstrated to be associated with a poor prognosis; in particular, a selective combination of C-reactive protein (CRP) and albumin (termed the modified Glasgow Prognostic Score, mGPS) has been shown to be of prognostic value in lung cancer 10. With respect to hematologic factors, a combination of the neutrophil-lymphocyte ratio (NLR) and the platelet-lymphocyte ratio (PLR) has been used to divide patients with advanced NSCLC into three different prognostic groups, prior to the initiation of treatment 11.

The presence of thrombocytosis can predict prognosis in many types of cancer, such as esophageal cancer 12 , gastric cancer 13, colorectal cancer 14, and NSCLC 15. Mean platelet volume (MPV) is an important indicator of platelet activation 16. Recent studies have revealed that MPV levels are abnormal in patients with NSCLC 16. It has been suggested that platelet count and its parameters, including plateletcrit, platelet distribution width (PDW), and MPV might be useful in combination with other acute phase reactants to define inflammation activation 17. Platelet parameters can be obtained using routine hematology analyzers during peripheral blood sampling. In comparison with other prognostic factors, platelet parameters are less costly and therefore can be used more extensively 16. To the best of our knowledge, few platelet parameters have been studied in cases of operable NSCLC, with the exception of platelet count and MPV. Therefore, the aim of the present study was to evaluate the clinical utility of platelet parameters in patients with NSCLC who underwent curative resection.

## Methods

### Patient selection and study design

A retrospective study was conducted on patients with NSCLC who underwent curative resection at the Kyung Hee University Hospital at Gangdong from July 2006 to September 2016. The study was approved by the Institutional Review Board of the Kyung Hee University Hospital at Gangdong (KHNMC 2017-01-004-001). Written informed consent was waived for this study, because of its retrospective nature. The electronic medical records of all patients with NSCLC who underwent curative resection were reviewed. The inclusion criteria were as follows: (i) patients who were diagnosed with primary NSCLC by expert pathologists, according to the 2015 World Health Organization (WHO) classification of lung tumors 18, and staged as I to IIIA, according to the 7th edition of the TNM Classification (Union for International Cancer Control); (ii) histological types of NSCLC, such as squamous cell carcinoma, adenocarcinoma, large cell carcinoma, or adenosquamous carcinoma; and (iii) patients who underwent pneumonectomy, bilobectomy, lobectomy, or segmentectomy. The exclusion criteria for patients were as follows: (i) patients who underwent preoperative chemotherapy or radiotherapy; (ii) presence of histologically confirmed neuroendocrine tumor, sarcomatoid carcinoma, small cell lung cancer (SCLC), or salivary gland tumor; (iii) history of liver cirrhosis (LC) or immune thrombocytopenic purpura (ITP); (iv) use of medications that may affect hemostasis; and (v) patients with stage IIIB or IV disease. A total of 228 patients were initially enrolled, and 112 patients were excluded for the following reasons: (i) 41 patients underwent wedge resection for diagnosis or palliation therapy; (ii) 12 patients had been diagnosed with neuroendocrine tumor; (iii) 8 patients were diagnosed with sarcomatoid carcinoma, 6 with SCLC, and 1 with a salivary gland tumor; (iv) 5 patients had undergone preoperative chemotherapy or radiotherapy; (v) 2 patients had LC and one had ITP; (vi) 32 patients were using aspirin, clopidogrel, cilostazol, or sarpogrelate; and (vii) 2 patients had stage IIIB disease and 2 had stage IV. Finally, 116 patients with NSCLC were included in the present study (supplementary figure 1). Computed tomography (CT) scans of the chest, abdomen, and pelvis, and positron emission tomography (PET)/CT scans were used for staging. Magnetic resonance imaging (MRI) or CT scans were used to evaluate potential brain metastases.

### Clinical variables

Records of clinical variables, such as sex, age, smoking status, histological type, histological grading, TNM stage, ECOG performance status, adjuvant therapy, maximal tumor dimension, and surgical resection type, were collected and analyzed. All laboratory data for patients were recorded before the patients underwent surgery. Data on blood tests obtained on the closest date to the day of surgery, within 7 days prior to surgery, were selected. Leukocytosis was defined as WBC counts higher than 11,000/µL. The diagnoses of anemia in men and women were based on hemoglobin levels less than 13 g/dL and 12 g/dL, respectively. The platelet parameters considered were platelet counts (PLT), MPV, PDW, and plateletcrit 19. Thrombocytosis was defined as platelet counts greater than 300 x 10^3^/µL.

### Optimum cut-off values

The prognostic impact of a continuous variable (e.g. plateletcrit) on the survival of patients with NSCLC was evaluated with maximally selected rank statistics using R (maxstat package) software. The recommended cut-off values of plateletcrit was 0.2755% for survivals in patients with NSCLC. To determine the most favorable cut-off values for clinical variables, receiver operating characteristic (ROC) curve analysis was used. The recommended cut-off values were as follows: MPV, 8.6 fL (sensitivity 90.3%, specificity 61.2%); area under the curve (AUC), 0.77. For the maximal tumor dimension, the cut-off value was 2.9 cm (sensitivity 83.9%, specificity 52.9%); AUC, 0.715.

### Statistical analysis

R software (version 3.2.3, R for Statistics Computing, Vienna, Austria) was used for statistical processing. OS was defined as the period from the date of surgery to the date of death. Data were represented as means ± standard deviations. Baseline clinical characteristics were compared using chi-squared tests for categorical values, and t-tests for continuous variables. The Cox proportional hazard regression model was used to evaluate the variables that demonstrated effects on OS; multivariate analysis was performed on these variables, with a P value of less than 0.05. The Cox proportional hazard regression model with backward elimination was used to validate the independent prognostic factors. Survival differences were analyzed using Kaplan-Meier analysis and the log-rank test. P values less than 0.05 were considered statistically significant.

## Results

### Baseline clinical characteristics of patients

A total of 116 patients with NSCLC were analyzed (supplementary figure 1). These patients were divided into two groups, based on their plateletcrit values; 15 patients had plateletcrit values greater than 0.2755%, and 101 had plateletcrit values of 0.2755% or less. Sex, age, histologic grading, smoking history, adjuvant therapy, TNM stage, maximal tumor dimension, Eastern Cooperative Oncology Group (ECOG) performance status, white blood cell (WBC) counts, hemoglobin counts, and MPV did not significantly differ between the two groups (Table 1). There was a significant increase in platelets in the patients with plateletcrit greater than 0.2755%. As shown in Table 1, 57 (49.1%) patients were aged 65 years or older, and 73 (62.9%) of the 116 patients were men. The most common histological subtype of NSCLC was adenocarcinoma, followed by squamous cell carcinoma, adenosquamous cell carcinoma, and large cell carcinoma. A total of 69 (59.5%) patients were categorized as having stage I disease, and 47 (40.5%) as stage II or IIIA. The ECOG performance status of all patients was either 0 (54.3%) or 1 (45.7%). Of the 116 patients, 47 (40.5%) had never smoked, and 42 (36.2%) had received adjuvant therapy, such as chemotherapy, radiotherapy, or chemoradiotherapy. Commonly used chemotherapy regimens were cisplatin plus vinorelbine (n = 17), and carboplatin plus paclitaxel (n = 14). The most common surgical modality was lobectomy, which was performed on 94 patients (81.0%), followed by segmentectomy, which was performed on 14 patients (12.1%).

### Relationship between platelet parameters and OS

**Figure 1:**
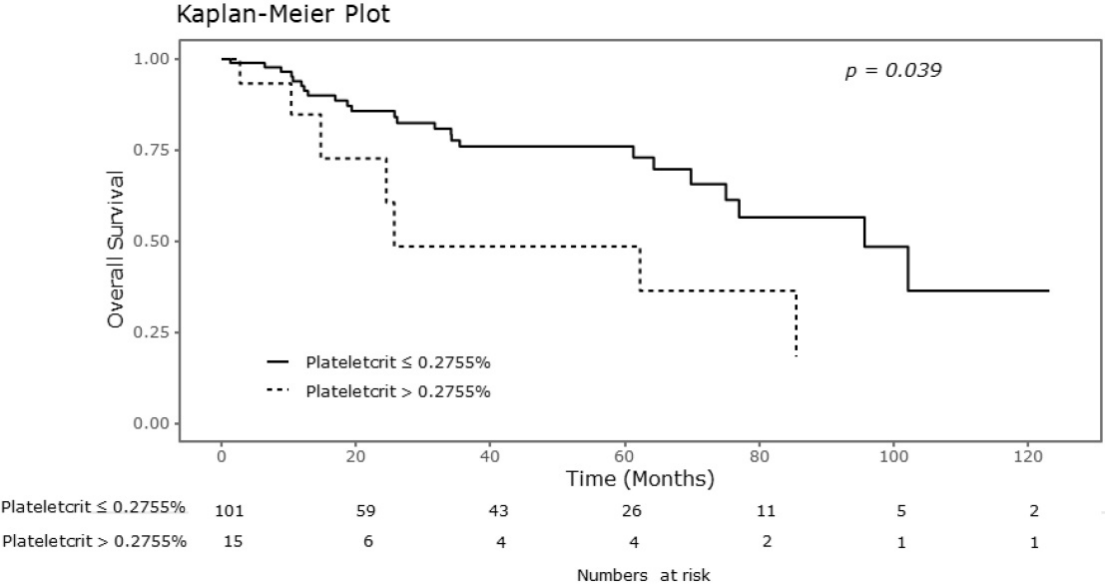
Kaplan-Meier curve for overall survival according to plateletcrit.

Significant differences were observed in the Kaplan-Meier survival curves for OS between the groups showing normal platelet counts and thrombocytosis. As shown in Table 2, the 5-year OS rates in the groups showing thrombocytosis and normal platelet counts were 0.526 and 0.810, respectively (P = 0.031). There were also significant differences in the overall survival curves with respect to plateletcrit (Figure 1). The 5-year OS rate was 0.899 in the group with MPV greater than 8.6 fL, and 0.683 in the group with MPV of 8.6 fL or less (P = 0.039). Additionally, the 5-year OS rates in the groups with plateletcrit of 0.2755% or less and greater than 0.2755% were reported as 0.761 and 0.485, respectively (P =0.039).

### Prognostic factors associated with OS

Univariate and multivariate analyses for OS are shown in Table 3. Univariate analyses identified male sex (P = 0.020), age 65 years or greater (P = 0.011), history of smoking (P = 0.012), non-adenocarcinoma histological type (P < 0.001), ECOG performance status of 1 (P = 0.008), maximal tumor dimension greater than 2.9 cm (P = 0.009), stage II or IIIA disease (P = 0.022), leukocytosis (P = 0.003), thrombocytosis (P = 0.035) and plateletcrit greater than 0.2755% (P = 0.046) as factors for OS that predicted poor prognosis. Multivariate analysis identified plateletcrit higher than 0.2755% (hazard ratio [HR] = 4.18, 95% confidence interval [CI] = 1.54-11.34, P =0.004), patient age of 65 years or more (HR = 4.02, 95% CI = 1.67-9.66, P = 0.001), and stage II or IIIA disease (HR = 2.95, 95% CI = 1.26-6.87, P = 0.012) as independent factors for OS that predicted a poor prognosis.

### Relationship between platelet parameters and DFS

**Figure 2:**
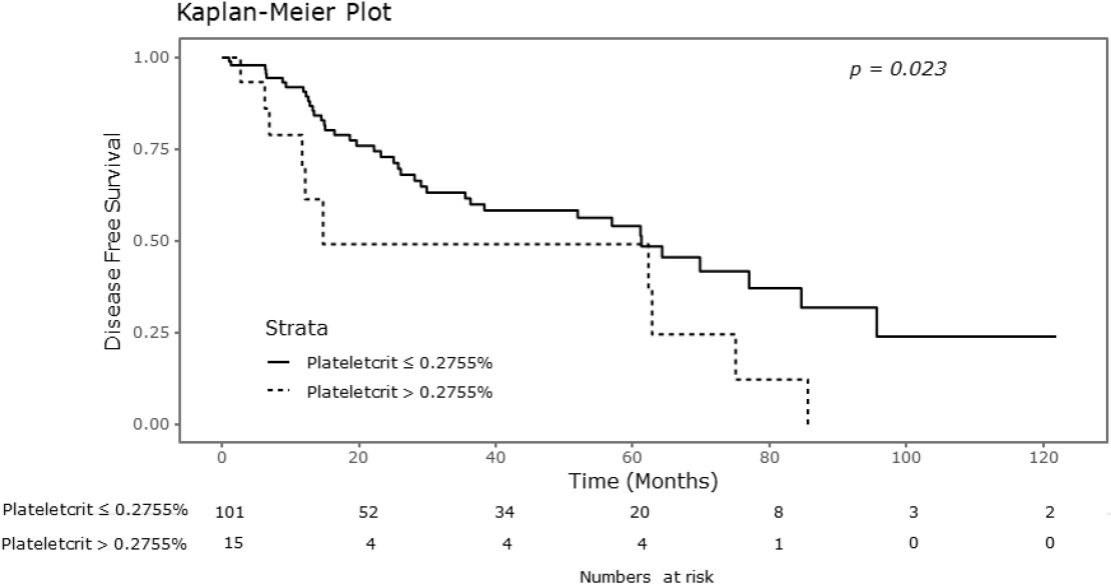
Kaplan-Meier curve for disease free survival according to plateletcrit.

Significant differences were observed in the Kaplan-Meier survival curves for DFS with respect to plateletcrit values, as shown in Figure 2. As shown in Table 2, the 5-year DFS rate was 0.541 in the group of patients with plateletcrit of 0.2755% or lower, compared to 0.491 in the group with plateletcrit greater than 0.2755% (P = 0.023). However, there were no significant differences in the DFS rates with respect to MPV and platelet counts.

### Prognostic factors associated with DFS

Univariate and multivariate analyses for DFS are shown in Table 4. Univariate analysis identified non-adenocarcinoma histological type (P = 0.001), histological grade 3 (P = 0.034), ECOG performance status of 1 (P = 0.003), maximal tumor dimension of more than 2.9 cm (P = 0.002), stage II or IIIA disease (P < 0.001), leukocytosis (P = 0.020), and plateletcrit greater than 0.2755% (P = 0.028) as factors for DFS that predicted poor prognosis. Multivariate analysis identified plateletcrit higher than 0.2755% (HR = 4.07, 95% CI = 1.52-10.94, P = 0.005), stage II or IIIA disease (HR = 5.38, 95% CI = 2.71-10.66, P < 0.001) and non-adenocarcinoma (HR = 1.92, 95% CI = 1.02-3.59, P = 0.040) as independent prognostic factors for DFS that predicted a poor prognosis.

## Discussion

Tumor-related humoral factors, such as granulocyte colony-stimulating factor (GCSF), interleukin-1 (IL-1), and interleukin-6 (IL-6), stimulate platelet production 20. Platelets release various cytokines, including vascular endothelial growth factor (VEGF) and platelet-derived growth factor (PDGF), which have significant roles in regulating angiogenesis 15. Experimental evidence suggests that platelets actively promote cancer progression through a variety of mechanisms, including protecting cancer cells from immune surveillance, negotiating the arrest of cancer cells in the microvasculature, and stimulating angiogenesis 15, 21. Decreased platelet surface expression of P-selectin and activated glycoprotein (GP)IIb/IIIa, *in vivo* and in response to protease-activated receptor (PAR)-1, PAR-4, and GP VI activation, has been associated with poor OS in patients with cancer 22.

The results of the present study indicate that preoperative plateletcrit may be used as a biomarker to predict outcomes in patients with NSCLC who undergo curative resections; this conclusion is based on the significant differences in DFS and OS observed in these patients, based on their plateletcrit values. It is expected that plateletcrit greater than 0.2755% would be associated with increased risks of death and disease recurrence. To the best of our knowledge, this study is the first to analyze the relationship between plateletcrit and prognosis in patients with NSCLC.

Plateletcrit refers to the volume occupied by the platelets in the blood 23 and is a marker of total platelet mass. The plateletcrit is determined by the formula platelet count times MPV divided by 10^4^, and the normal range for plateletcrit is 0.22-0.24% 23. In a study of patients with papillary thyroid carcinoma (PTC), the plateletcrit ranges were 0.24±0.05 in the group with PTC and 0.17±0.02 in the control group, with a significant difference between the two groups (P < 0.01) 24. In the present study, the plateletcrit range was 0.0870-0.3802% and the cut-off value was 0.2755% according to maximally selected rank statistics using R (maxstat package) software. However, data investigating the importance of plateletcrit among platelet parameters are limited 25.

The reason for the association between elevated plateletcrit and worse outcomes for patients with NSCLC remains unknown. However, plateletcrit is also related to platelet activation 17. Plateletcrit has been used as a predictive marker in the discrimination of autoimmune gastritis 26, and it is an important predictor for saphenous vein graft disease (SVGD) 27. Plateletcrit may act as a sensitive and specific biomarker for determining disease activity in Crohn's disease, especially in those with high-sensitivity CRP (hs-CRP) lower than 10.0 mg/L 28. Significantly, an association between high plateletcrit and poor OS in patients with locally advanced pancreatic adenocarcinoma has been reported 17. In addition, plateletcrit was shown to be consistently higher in a group of patients with epithelial ovarian cancer (EOC) than in the benign tumor and healthy groups, and the trend toward higher levels of platelet parameters reflected enhancement of bone marrow hematopoietic activity 29.

The present study has several limitations. First, as this is the first report on the clinical utility of plateletcrit in patients with lung cancer, the optimal cut-off values for plateletcrit are yet to be defined. Therefore, the results of this study need to be interpreted with caution. Second, among the platelet parameters, the PDW was not analyzed because data pertaining to PDW could not be obtained. The PDW indicates volume variability in the size of platelets, and is a valuable marker for platelet activity; thus, in further studies, the PDW must be included in the analysis. In conclusion, our results suggest a potential role of preoperative plateletcrit as an independent prognostic marker for patients with resectable NSCLC. However, the results obtained from our study are hypothesis generating, and should be confirmed with larger studies. In addition, internal and external validation will be needed in future prospective studies to rule out chance findings.

## Supplementary Material

Supplementary Figure 1.

## Authors' Contributions

Joon Young Hur and Wan Kyu Eo contributed to the study conception and design. Joon Young Hur, Ha Yeon Lee and Hye Jung Chang contributed to revision of the manuscript. Joon Young Hur contributed to acquisition of data and drafting of manuscript. Dae Hyun Kim and Chun Woong Choi analyzed the manuscript. All authors read and approved the final manuscript.

## Figures and Tables

**Table 1:** Baseline characteristics of NSCLC patients.

Variables	Plateletcrit ≤ 0.2755% (n=101)	Plateletcrit > 0.2755% (n=15)	Total(n=116)	*P*
Sex				0.544
Female	39 (38.6%)	4 (26.7%)	43 (37.1%)	
Male	62 (61.4%)	11 (73.3%)	73 (62.9%)	
Age (years)				0.630
< 65	50 (49.5%)	9 (60.0%)	59 (50.9%)	
≥ 65	51 (50.5%)	6 (40.0%)	57 (49.1%)	
Histologic type				0.009
ADC	72 (71.3%)	5 (33.3%)	77 (66.4%)	
Non-ADC	29 (28.7%)	10 (66.7%)	39 (33.6%)	
Histologic grade				0.680
G1/G2	79 (78.2%)	13 (86.7%)	92 (79.3%)	
G3	22 (21.8%)	2 (13.3%)	24 (20.7%)	
Smoking				0.745
Never	42 (41.6%)	5 (33.3%)	47 (40.5%)	
Past or current	59 (58.4%)	10 (66.7%)	69 (59.5%)	
ECOG PS				0.719
0	56 (55.4%)	7 (46.7%)	63 (54.3%)	
1	45 (44.6%)	8 (53.3%)	53 (45.7%)	
Adjuvant therapy				0.968
No	65 (64.4%)	9 (60.0%)	74 (63.8%)	
Yes	36 (35.6%)	6 (40.0%)	42 (36.2%)	
Maximal dimension of tumor (cm)				1.000
< 2.9	43 (42.6%)	6 (40.0%)	49 (42.2%)	
≥ 2.9	58 (57.4%)	9 (60.0%)	67 (57.8%)	
Stage				0.325
I	60 (59.4%)	9 (60.0%)	69 (59.5%)	
II or IIIA	41 (40.6%)	6 (40.0%)	47 (40.5%)	
Leukocytosis				0.136
No	99 (98.0%)	13 (86.7%)	112 (96.6%)	
Yes	2 (2.0%)	2 (13.3%)	4 (3.4%)	
Anemia				0.340
No	64 (63.4%)	7 (46.7%)	71 (61.2%)	
Yes	37 (36.6%)	8 (53.3%)	45 (38.8%)	
Thrombocytosis				<0.001
No	86 (85.1%)	3 (20.0%)	89 (76.7%)	
Yes	15 (14.9%)	12 (80.0%)	27 (23.3%)	
MPV (fL)				0.774
≤ 8.6	55 (54.5%)	7 (46.7%)	62 (53.4%)	
> 8.6	46 (45.5%)	8 (53.3%)	54 (46.6%)	
Operation				0.272
Bilobectomy	3 ( 2.8%)	2 (13.3%)	5 ( 4.3%)	
Lobectomy	83 (82.2%)	11 (73.3%)	94 (81.0%)	
Pneumonectomy	2 ( 2.0%)	1 (6.7%)	3 ( 2.6%)	
Segmentectomy	13 (12.9%)	1 (6.7%)	14 (12.1%)	

ADC: adenocarcinoma; ECOG PS: eastern cooperative oncology group performance status; WBC: white blood cell; Hb: hemoglobin; MPV: mean platelet volume.

**Table 2:** Analysis for 5 year overall survival and 5 year disease free survival.

Variables	5YR OS	*P*	5YR DFS	*P*
Sex		0.015		0.355
Female	0.902		0.643	
Male	0.631		0.473	
Age (years)		0.008		0.600
< 65	0.845		0.555	
≥ 65	0.619		0.513	
Histologic type		<0.001		<0.001
ADC	0.858		0.651	
Non-ADC	0.500		0.336	
Histologic grade		0.412		0.030
G1/G2	0.765		0.576	
G3	0.535		0.357	
Smoking		0.008		0.093
Never	0.912		0.688	
Past or current	0.606		0.427	
ECOG PS		0.005		0.002
0	0.831		0.631	
1	0.619		0.431	
Maximal dimension of tumor (cm)		0.005		0.001
< 2.9	0.940		0.756	
≥ 2.9	0.593		0.388	
Stage		0.019		<0.001
I	0.908		0.834	
II or IIIA	0.530		0.201	
Leukocytosis		<0.001		0.011
No	0.756		0.553	
Yes	0		0	
Anemia		0.354		0.097
No	0.748		0.599	
Yes	0.699		0.450	
Thrombocytosis		0.031		0.191
No	0.810		0.559	
Yes	0.526		0.463	
MPV (fL)		0.039		0.437
> 8.6	0.899		0.537	
≤ 8.6	0.683		0.524	
Plateletcrit (%)		0.039		0.023
≤ 0.2755	0.761		0.541	
> 0.2755	0.485		0.491	

OS, overall survival; DFS, disease free survival; ADC, adenocarcinoma; ECOG PS, eastern cooperative oncology group performance status; MPV, mean platelet volume.

**Table 3:** Analysis of prognostic factors for overall survival.

Variables		Univariate			Multivariate	
	HR	95% CI	*P*	HR	95% CI	*P*
Sex : Female vs. male	3.12	1.19-8.15	0.020	-	-	*-*
Age (years): <65 vs. ≥65	2.83	1.26-6.33	0.011	4.02	1.67-9.66	0.001
Histologic type : ADC vs. non-ADC	3.93	1.88-8.23	<0.001	*-*	*-*	-
Histologic grading : G1 or G2 vs. G3	1.46	0.59-3.60	0.414	*-*	*-*	-
Smoking : never vs. past or current	3.14	1.29-7.67	0.012	*-*	*-*	-
ECOG PS : 0 vs. 1	2.82	1.32-6.03	0.008	*-*	*-*	-
Maximal dimension of tumor (cm) : < 2.9 vs. ≥ 2.9	3.58	1.37-9.36	0.009	*-*	*-*	-
Stage : I vs. II or IIIA	2.32	1.13-4.80	0.022	2.95	1.26-6.87	0.012
Leukocytosis : No vs. Yes	6.50	1.89-22.46	0.003	*-*	*-*	-
Anemia : No vs. Yes	1.40	0.68-2.87	0.356	*-*	*-*	-
Thrombocytosis : No vs. Yes	2.18	1.06-4.51	0.035	*-*	*-*	-
MPV (fL) : > 8.6 vs. ≤ 8.6	0.30	0.90-1.01	0.052	*-*	*-*	-
Plateletcrit (%): ≤ 0.2755 vs. > 0.2755	2.37	1.01-5.54	0.046	4.18	1.54-11.34	0.004

HR, hazard ratio; CI, confidence interval; ADC, adenocarcinoma; MPV, mean platelet volume.

**Table 4:** Analysis of prognostic factors for disease free survival.

Variables		Univariate			Multivariate	
	HR	95% Cl	*P*	HR	95% CI	*P*
Sex : Female vs. male	1.35	0.73-2.49	0.346	-	*-*	*-*
Age : <65 vs. ≥65	1.16	0.66-2.06	0.601	*-*	*-*	*-*
Histoligic type : ADC vs. non-ADC	2.55	1.45-4.48	0.001	1.92	*1.02-3.59*	0.040
Histologic grading : G1 or G2 vs. G3	2.11	1.06-4.23	0.034	*-*	*-*	*-*
Smoking : never vs. past or current	1.67	0.91-3.04	0.097	*-*	*-*	*-*
ECOG PS : 0 vs. 1	2.45	1.36-4.43	0.003	*-*	*-*	*-*
Maximal dimension of tumor (cm) : < 2.9 vs. ≥ 2.9	2.84	1.45-5.58	0.002	*-*	*-*	*-*
Stage : I vs. II or IIIA	4.56	2.48-8.38	<0.001	5.38	2.71-10.66	<0.001
Leukocytosis : No vs. Yes	4.12	1.25-13.61	0.020	*-*	*-*	*-*
Anemia : No vs. Yes	1.60	0.91-2.81	0.101	*-*	*-*	*-*
Thrombocytosis : No vs. Yes	1.48	0.82-2.66	0.194	*-*	*-*	*-*
MPV (fL) : > 8.6 vs. ≤ 8.6	0.76	0.39-1.51	0.438	*-*	*-*	*-*
Plateletcrit (%) : ≤ 0.2755 vs. > 0.2755	2.20	1.09-4.45	0.028	4.07	1.52-10.94	0.005

HR, hazard ratio; CI, confidence interval; ADC, adenocarcinoma; MPV, mean platelet volume.

## Abbreviations

ADC: adenocarcinoma
ECOG PS: eastern cooperative oncology group performance status
WBC: white blood cell
Hb: hemoglobin
MPV: mean platelet volume
OS: overall survival
DFS: disease free survival
HR: hazard ratio
CI: confidence interval
NSCLC: Non-small cell lung cancer
SCLC: small cell lung cancer
ITP: immune thrombocytopenic purpura
EGFR: epidermal growth factor receptor
ALK: anaplastic lymphoma kinase
TKI: tyrosine kinase inhibitors
PD-L1: programmed death ligand 1
SIR: systemic inflammatory response
CRP: C-reactive protein
mGPS: modified Glasgow Prognostic Score
NLR: neutrophil-lymphocyte ratio
PLR: platelet-lymphocyte ratio
PDW: platelet distribution width
ROC: receiver operating characteristic
AUC: area under the curve
