# Supplementary figures and images for: Preoperative plateletcrit is a Prognostic Biomarker for Survival in Patients with Non-Small Cell Lung Cancer
Source: J Cancer. 2020 Feb 25;11(10):2800–7. doi: 10.7150/jca.41122 (PMC7086273; doi:10.7150/jca.41122)

Supplementary Figure 1. Flow chart of patient selection

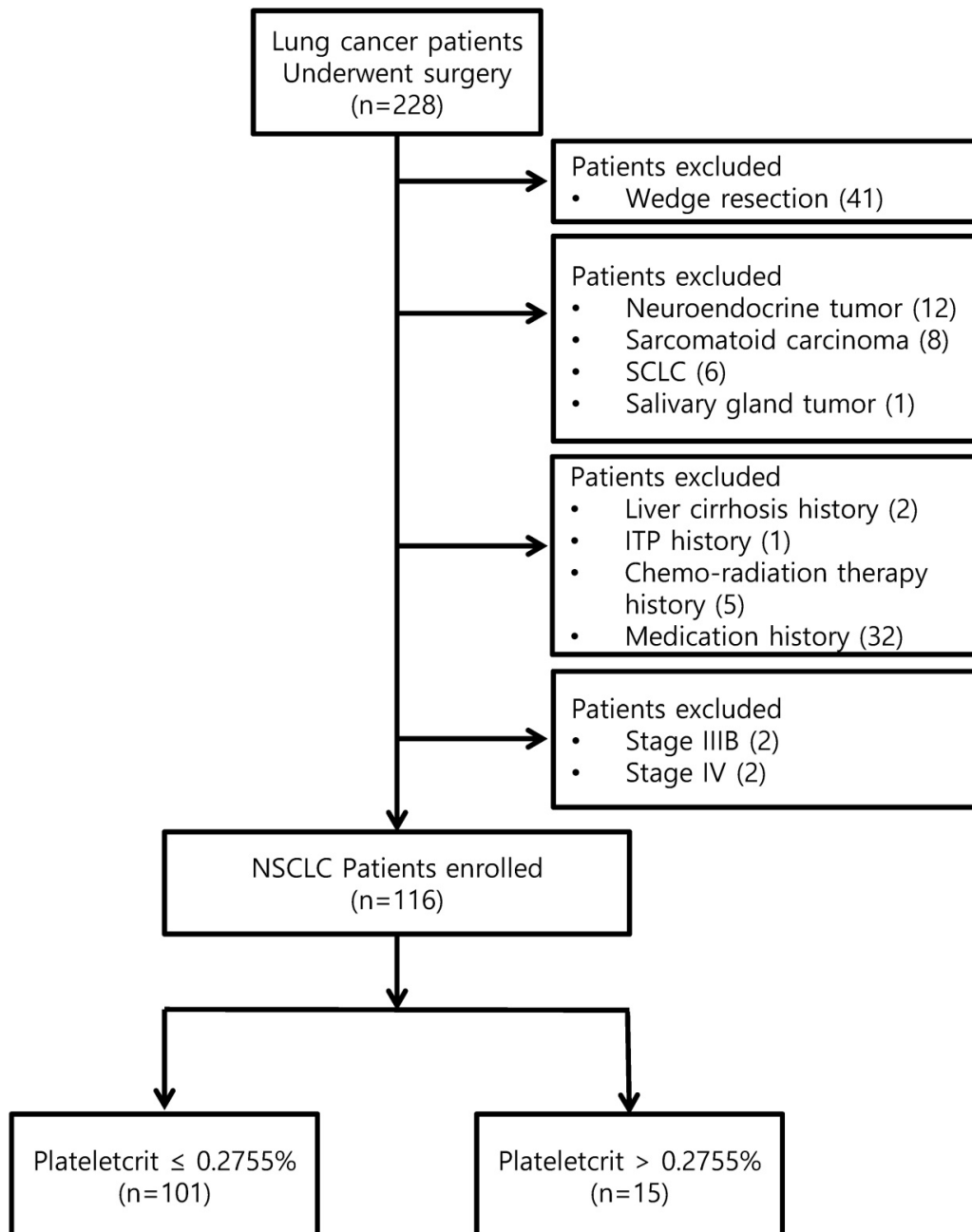

Supplement: Supplementary file 1 — Supplementary Figure 1. [file jcav11p2800s1.pdf]
